# P-1383. Proapoptotic Bcl-2 Inhibitors as Adjunctive Host-Directed Therapy Enhance Tuberculosis Treatments and Long-term Cure in a Murine Model

**DOI:** 10.1093/ofid/ofaf695.1570

**Published:** 2026-01-11

**Authors:** Medha Singh, Mona O Sarhar, Oscar J Nino-Meza, Yuderleys Masias-Leon, Eric O Aboagye, Laurence S Carroll, Sanjay K Jain

**Affiliations:** Johns Hopkins University, Baltimore, MD; Johns Hopkins University, Baltimore, MD; Johns Hopkins University School of Medicine, Baltimore, Maryland; Johns Hopkins University School of Medicine, Baltimore, Maryland, United States., Baltimore, MD; Imperial college, London, London, England, United Kingdom; Johns Hopkins University, Baltimore, MD; Johns Hopkins University, Baltimore, MD

## Abstract

**Background:**

*Mycobacterium tuberculosis* evades the host by upregulating anti-apoptotic Bcl-2 family proteins causing necrosis, and fibrosis that hinder immune responses and antibiotic penetration. We recently reported that navitoclax, an orally bioavailable proapoptotic Bcl-2 inhibitor, administered at human-equipotent doses in a mouse model of pulmonary tuberculosis (TB), improved bacterial clearance, reduced extrapulmonary dissemination, and limited pulmonary necrosis and fibrosis. Based on these findings, we hypothesized that navitoclax may help eliminate persister bacilli and improve relapse-free cure rates when used adjunctively with standard TB treatment.

**Methods:**

To test this hypothesis, we assessed relapse rates *in M. tuberculosis*-infected mice treated with standard TB treatment (rifampin – R, isoniazid – H and pyrazinamide – Z, RHZ) with or without navitoclax (Fig. 1a), all dosed orally at human equipotent levels (Fig. 1b). Relapse was assessed 16 weeks after cessation of 12 weeks of treatments. We also performed PET imaging in *live* animals using custom built biosafety-compliant imaging cells with ^18^F-ICMT-11 (targeting activated caspase 3/7) and ^18^F-FAPI-74 (targeting fibroblast activation protein inhibitor) for these set of studies.

**Results:**

The bacterial implantation and response to treatment demonstrated that the addition of navitoclax to the standard TB treatment decreased the pulmonary bacterial burden (Fig. 1c). Importantly, the addition of navitoclax to the standard TB treatment, reduced the relapse rate from 83.3% (25 out of 30 animals) to 47.5% (19 out of 40 animals; *P* < 0.01 using single tailed chi square test) (Fig. 2). Imaging showed significantly higher pulmonary tissue apoptosis, quantified using ^18^F-ICMT-11 PET (Fig. 3, *P* = 0.04) and significantly reduced fibrosis quantified using ^18^F-FAPI-74 PET (Fig. 4, *P* < 0.01) in animals receiving adjunctive navitoclax versus those receiving standard TB treatment alone.

**Conclusion:**

Our studies suggest that proapoptotic drugs can improve pulmonary TB treatments, reduce lung damage / fibrosis and may be protective against post-TB lung disease. Imaging with clinically translatable biomarkers for apoptosis and fibrosis could help characterize post-TB lung disease as well as evaluate HDTs in early clinical trials.

**Disclosures:**

All Authors: No reported disclosures

